# The impact of Covid-19 pandemic on services for children and adolescents with ADHD: results from a survey of paediatricians in the United Kingdom

**DOI:** 10.3934/publichealth.2022037

**Published:** 2022-06-21

**Authors:** Michael O Ogundele, Hani F Ayyash, Cornelius Ani

**Affiliations:** 1 Halton Community Pediatrics Unit, Bridgewater Community Healthcare NHS Foundation Trust, Runcorn UK; 2 Essex Partnership University Hospitals NHS Foundation Trust (EPUT), Lighthouse Child Development Centre, Southend-On-Sea, Essex, UK; 3 Division of Psychiatry, Imperial College London, UK; 4 Surrey and Borders Partnership NHS Foundation Trust; 5 Scientific Committee Member, British Paediatric Surveillance Unit, Royal College of Paediatrics and Child Health, UK; 6 George Still Forum (National Network for Paediatric ADHD and related Neurodevelopmental disorders)

**Keywords:** Covid-19, ADHD, service provision, disruption, child and adolescent mental health, paediatrics, telemedicine, United Kingdom

## Abstract

**Background:**

The Covid-19 pandemic has led to huge disruptions and multi-domain healthcare crisis, with additional impact on children and young people (CYP) affected by Attention Deficit and Hyperactivity Disorder (ADHD).

**Methods:**

We conducted an online survey and obtained responses from 62 Paediatricians who provide ADHD services for CYP about their experience of Service disruption and adaptations during the first Covid-19 lockdown in the United Kingdom between March and June 2020. The responses were both quantitative and qualitative.

**Results:**

The Paediatricians reported huge service disruptions such that almost half ceased the assessment of new patients with ADHD, and only 5% were able to offer physical monitoring for most patients. However, all respondents had adopted telemedicine, which allowed them to maintain high levels of non-physical service provision for existing patients. The Paediatricians used risk stratification strategies to determine which patients were more likely to benefit from the limited available face to face appointments for physical monitoring. The Paediatricians demonstrated clinical pragmatism to meet the needs of their patients such as starting medication without physical exam especially if the patient's behaviour was so challenging that it was presenting a crisis at home, and setting aside monthly limits for stimulant medications. Some respondents reported helpful cross-service collaborations to support CYP with ADHD and their families.

**Conclusion:**

The Covid-19 pandemic has had adverse effect on many CYP with ADHD and caused huge disruption to the ADHD services that support them. As the pandemic continues to cause disruptions to ADHD services, the service adaptations emerging from the literature including some of those identified in this study could be useful to support more stable and sustainable ADHD services, both during and after the pandemic.

## 1. Introduction

The Covid-19 pandemic has led to huge multidimensional disruptions to societal function across the world [1]. The disruption has been associated with multi-domain healthcare crisis [2]. Healthcare facilities have been overstretched in many countries due to the unprecedented medical demands to treat large numbers of people infected by Covid-19. Furthermore, the mental health of populations have suffered from direct impact of Covid-19 and the unintended consequences of lockdown and social distancing measures to control the pandemic [1]. Recognition of the mental health impact of Covid-19 has led to calls to integrate mental health into Covid-19 response [3]. A particular concern is the adverse impact of Covid-19 on children and young people (CYP) affected by Attention Deficit and Hyperactivity Disorder (ADHD) [4].

ADHD is a common neurodevelopmental condition affecting 5% of school aged children [5]. The condition is characterized by functionally impairing levels of inattention, hyperactivity, and impulsivity [6]. ADHD is one of the commonest conditions treated in specialist Child and Adolescent Mental Health services (CAMHS) [7], and increasingly by Community Child Health paediatric services [8]. It is hypothesised that the core features of ADHD can be particularly challenging in dealing with Covid-19 infection [9]. For example, symptoms such as not paying attention, not following instructions, being easily distractible, restlessness, and impulsiveness could increase the risk of exposure to Covid-19 by making it harder for people with ADHD to abide by typical Covid-19 infection control strategies such as frequent hand washing, social distancing, wearing face mask consistently and isolating when required to [2]. This hypothesis is now supported by studies showing an association between ADHD symptoms and increased likelihood of non-adherence to Covid-19 safety measures [10], and increased Covid-19 infection rates in people with untreated ADHD compared with those without ADHD, and persons with ADHD who are on medication [11]. In one study, the presence of ADHD was a strong predictor for COVID illness similar to other more well-known medical risk factors such as diabetes mellitus, cardiovascular diseases, and obesity [2].

The increased association between untreated ADHD and increased risk profile from Covid-19 infection, highlights the importance of sustaining ADHD services during the pandemic. This is to ensure that affected persons continue to access adequate ADHD-related care; which could reduce their risk of Covid-19 infection. Unfortunately, ADHD services were not spared by the massive disruption engendered by the pandemic and related lockdowns [12]. The Covid-19 disruption led to significant difficulties for affected children and young people and their families [13]. Studies have shown that some CYP experienced increased ADHD symptoms and other behavioural difficulties during the pandemic compared with pre-pandemic [14],[15]. Access to ADHD services was more difficult especially following the first lockdowns as services adjusted to moving from face to face to remote provision [12]. Starting new medications was paused by many services as reliable feedback was difficult to receive and confounded by the lockdown [7]. Many services halted new assessments as supporting information from schools were difficult to access [7]. Referral rates for new ADHD assessments reduced [7] as schools which make or support a significant proportion of referrals were closed. On the other hand, after the lockdowns, the pent up demand for ADHD assessments and treatments have led to increased post-lockdown pressure on ADHD services and CAMHS [16]. This increased demand has exacerbated pre-existing pressures on CAMHS services, which already had long waiting times for assessments and treatment.

Recognising the potential protective role of ADHD treatment in reducing the risk of Covid-19 infection transmission [11], and the risk of service disruption from the pandemic, various authorities such as the European ADHD Guidelines Group issued pragmatic guidelines to support clinicians to adjust their practices in order to maintain service provision [17]. The adjustments included starting medication where physical assessment is not feasible provided that the young person's and their family's medical histories do not have specific indices suggestive of risk of cardiovascular disease [17]. As the pandemic progressed, services adapted ADHD assessment and treatment for remote delivery by telephone and or video, which studies show are effective [18],[19]. The limited opportunities for face to face appointment were prioritised for patients with specific need for physical assessment such as cardiovascular monitoring. However, there are concerns about the accuracy of in-person cardiovascular monitoring during the lockdown. For example, one study showed that CYP whose Blood Pressures were checked in the clinic during the lockdown tended to have elevated readings which returned to normal after the lockdown without further intervention [20]. Other adjustments made by ADHD services to reduce exposure to Covid-19 infection included minimising journeys to the clinics for medication prescriptions by posting prescriptions to families, and exceeding the 30 days limit for supply of controlled medications [7].

Two years into the pandemic, service disruptions continue to be caused by Covid-19. At the time of writing this paper, many countries were in their 4^th^ wave of Covid 19 infection and were re-introducing restrictions due to the rapid spread of the Omicron variant. Thus, there continues to be a need to reflect on the strategies to support ADHD services for providing sustainable care for CYP with ADHD while the pandemic continues. Some of the adjustments and lessons learnt will remain useful after the pandemic and help to inform future service adaptions due to as yet unknown challenges such as a new pandemic. The current study explored the Covid-19 pandemic experiences of United Kingdom (UK)-based Paediatricians who assess and treat CYP with ADHD. We sought the Paediatricians' views on Service disruptions and the adjustments they made during the first Covid-19 lockdown in the UK between March and June 2020. The findings of the study add to the increasing pool of studies helping to shape practice in the management of ADHD during the continuing Covid-19 pandemic and beyond.

## 2. Design and methods

An online custom-designed questionnaire was developed by two Consultant Neuro-developmental Paediatricians with further input from a Consultant Child and Adolescent Psychiatrist. The questionnaire included structured and open-ended questions related to practice during the first Covid-19 pandemic lockdown in United Kingdom between March and June 2020. The structured questions were: experience of redeployment to other clinical areas to support Covid-19 patients; proportion of new and follow-up patients seen for face to face and or remote consultations; proportion of follow-up patients having their height, weight and Blood Pressure monitored according to UK guidelines; experience of starting, titrating or changing medications with or without recent physical measurements; awareness of specific pandemic-related guidelines for ADHD management; forms of telemedicine or digital technology being used for patient consultation; other modifications or flexibilities introduced to usual clinical practice; use of non-pharmacologic support strategies, and involvement of other staff in supporting ADHD management. Each closed question was followed by an open-ended question which invited participants to provide free-text qualitative comments to contextualise their responses to the closed question. The survey was approved by the Executive committee of the George-Still Forum (GSF), which is a UK-wide ADHD Clinical Network whose membership consists of mainly Neurodevelopmental Paediatricians. Links to the survey were emailed to all the delegates who attended the GSF annual scientific meeting in 2019 and provided consent to be contacted. The survey ran between June and July 2020. Responses to structured questions are presented in the results as proportions. We applied reflexive thematic content analysis [21] to the free text comments based on inductive approach. Details of these procedures are available elsewhere [21] but in summary, we extracted the free text comments, familiarised ourselves with the content, identified codes, and examined the codes for common meanings, which we used to create themes. In line with current research governance in the UK and other countries in relation to low risk health and medical research [22], ethical approval was not required as the survey was entirely voluntary, and the link was sent only to participants who had given a priori consent to be contacted and not classified as “vulnerable”.

## 3. Results

A total of 62 responses were received out of 115 delegates who attended the last GSF annual scientific conference in 2019 who were sent the survey link by email. This represents 54% response rate. Most of the respondents (62%) were not redeployed from their main clinical roles to support Covid-19 patients. Consistent with the prevailing pandemic-induced change in service delivery, all respondents had adopted remote service provision, of which the most common was by telephone (98%). Report of service disruption was most pronounced for new ADHD referrals as almost half of respondents (47%) ceased service provision for this cohort and only 28% were able to maintain services for most of their new referrals. Conversely, service provision remained high for existing patients requiring non-physical ADHD follow-up appointments. For the latter cohort, 65% of respondents were still offering services to most patients, and only 16% interrupted their service provision.

The respondents' qualitative comments provided further insights into these altered service arrangements. For example, some respondents pointed out that they ceased new ADHD assessments because the simultaneous closure of schools meant that the collateral information required for completion of new ADHD assessments was not available. Others noted that where the collateral information was already available, they were able to proceed with new ADHD assessment through remote sessions. Similarly, some respondents felt able to remotely initiate medications for patients newly diagnosed with ADHD if the patient had already had normal physical exam prior to the lockdown.

The physical monitoring required for ADHD management was severely interrupted such that only 5% of respondents reported being able to obtain measurements for most of their patients. Where physical monitoring occurred, most still occurred in specialist centres (25%), while others took place at home (13%), or in primary care (10%). Qualitative comments indicated that clinicians used risk stratification strategy to determine the patients most likely to benefit from the limited available face to face appointments for physical monitoring. In this context, respondents prioritised face-to-face monitoring for: patients with pre-existing concern about their physical well-being such as weight already below 9^th^ centile, those on the upper ceiling of the normal range of medications (e.g., 2.1mg/kg of methylphenidate), patients who had had home measurements that were outside normal limits, or those with other presentations considered to require urgent face to face clinical review.

The disruption of face-to-face clinical contact and difficulty with obtaining physical measurements led some respondents to adopt more cautionary approaches to ADHD clinical work. Qualitative comments highlighted two such cautions: not to initiate new medications especially when baseline physical investigation was not possible, and avoiding changes to the dose of existing medication unless the patient was well known and had a trend from past physical measurements that was in the normal range.

Some of the participants were aware of the European ADHD Guidelines Group Covid-19 recommendation [17] that was published in the early stages of the pandemic. The Guideline acknowledged that there were no risk-free options in the pandemic; hence it provided clinically pragmatic approaches to service provision. This clinical pragmatism was evident in the survey as many respondents adopted practices that deviated from the “norm” but offered a justifiable balance of benefit over risks. Examples from qualitative comments include: starting medication without physical exam especially if the patient's behaviour was so challenging that it was presenting a crisis at home, carefully weighing the risk-benefit ratio of home visits and where necessary proceeding with appropriate personal protective equipment (PPE) and social distancing, setting aside monthly limit and prescribing two-months' worth of stimulant medications, and accepting physical measurements from home and school without insisting on calibration of the instruments.

The respondents reported how the alterations in clinical service provision during the pandemic led to promotion of self-reliance among families. The respondents indicated that at the beginning of the pandemic, many families were not able to check weight, height, blood pressure and height at home but the proportion increased during the lockdown. In some cases, specialist services sourced the equipment for families while clinicians provided written or live guidance on their appropriate use. Many families preferred home measurements due to worry about exposure to Covid-19 if they came to the clinic. Some respondents rationalised which measurements families needed to focus on. For example, some advised less focus on height because, in their experience, once yearly height measurements can be adequate.

Similarly, some respondents reported some helpful cross-service collaborations to support CYP with ADHD during the first Covid-19 pandemic lockdown in the UK. Respondents mentioned the support of Specialist Nurses who provided more than a quarter (26%) of the required face to face appointments. Some services set up specific nurse-led clinics for physical monitoring for patients who needed to be seen in person. Collaboration with clinicians in Child and Adolescent Mental health services (CAMHS) was also reported to have provided 19% of the required face to face appointments. Some schools supported CYP with ADHD by offering physical monitoring for the few more vulnerable children who were allowed to be in school during the lockdown.

## 4. Discussion

The Covid-19 pandemic caused significant challenges for CYP with ADHD and their families, and for the services that support them. This survey of Paediatricians providing ADHD assessment and treatment during the first Covid-19 lockdown in the UK between March and June 2020 noted huge service disruptions such that almost half of the respondents ceased the assessment of new patients with ADHD, and only 5% were able to offer physical monitoring for most patients. However, all respondents had adopted telemedicine, which allowed them to maintain high levels of non-physical service provision for existing patients. The Paediatricians used risk stratification strategies to determine which patients were more likely to benefit from the limited available face to face appointments for physical monitoring. The Paediatricians demonstrated clinical pragmatism to meet the needs of their patients such as starting medication without physical exam especially if the patient's behaviour was so challenging that it was presenting a crisis at home, and setting aside monthly limits for stimulant medications. Some respondents reported helpful cross-service collaborations to support CYP with ADHD and their families.

These findings are consistent with other studies from different parts of the world such as summarised in a recent review [12]. The review documented widespread disruption of ADHD services due to the Covid-19 pandemic. However, it also showed resilient responses by services as they made adjustments and adaptations to maintain ongoing care. A key adjustment was the widespread adoption of telemedicine-based service provision. Other important adaptations include more flexible approaches to the requirement for physical monitoring for ADHD treatment.

Some CYP with ADHD have experienced increased ADHD symptoms and related difficulties during the ongoing Covid-19 pandemic [14],[15]. Also, persons with ADHD are at increased risk of Covid-19 infection [23]. However, one positive note is that ADHD treatment can reduce the risk of Covid-19 infection [11]. Taken together, these findings show the importance of sustaining effective ADHD management during the Covid-19 pandemic in order to mitigate the more immediate risks related to Covid-19 infection. Furthermore, continuing ADHD treatment during the pandemic could help to reduce the well documented multiple adverse outcomes of untreated ADHD on the mental and physical health, education, and social domains of affected individuals [24]. Thus, it is important to identify the beneficial adjustments and adaptions helping to sustain ADHD services during the pandemic so that these can be maximised. Some of such adjustments highlighted by the current study include telemedicine, pragmatic application of existing evidence-based guidance and policies, using risk stratification to determine offer of face to face appointments, empowering families and other organisations to support off-site physical monitoring, and inter-service collaboration.

Telemedicine has been crucial in maintaining ADHD Service provision while limiting face-to-face contact to reduce the risk of Covid-19 transmission. There is increasing evidence that tele-medicine is effective in the diagnosis and treatment of ADHD [18],[19]. ADHD may be particularly suited to telemedicine because the assessment and treatment processes can be well structured. Also both medical and psychological interventions for ADHD are amenable to remote delivery. For example, ADHD-related psycho-education and parent training programmes are deliverable by video [25].

One of the challenges for telemedicine-based ADHD treatment is the need for physical examination. The current study and others show that some parents were able to check weight, height, Blood Pressure, and Pulse at home. Some ADHD services are providing the equipment for families that do not already have them. Some clinicians in the current study were able to provide live guidance for parents to use the equipment. Also there are easy to access online instructions on how to check Blood Pressure (https://www.bhf.org.uk/informationsupport/support/manage-your-blood-pressure-at-home). One advantage of home Blood Pressure monitoring is reduced potential for “white coat syndrome”. For example, one study showed that CYP with ADHD seen face to face during the first Covid-19 lockdown in the UK tended to have higher Blood Pressure compared with their re-pandemic readings but improved after the lockdown without further intervention [20]. In the meantime, where measurement of Blood Pressure and Pulse is not feasible due to the pandemic, evidence-based guidelines suggest proceeding with treatment if none of specific adverse personal or family cardiovascular histories applied to the patient [17]. In addition to ADHD assessment and treatment in the context of Covid-19, telemedicine has other advantages [26] such as reduced traveling times for clinicians and families. The time saving can improve clinicians' productivity, which can be useful in managing the increase in post-lockdown referral rates for ADHD assessment and treatment. Telemedicine also reduces the need for CYP to take time off school or for their parents to take time off work. It can also reduce travel cost for families, and help the environment. Maximising these benefits of telemedicine could be one of the positive lessons from the Covid-19 pandemic in relation to the assessment and treatment of CYP with ADHD.

While acknowledging the important contribution of telemedicine, the nature of ADHD service provision is such that some face to face meetings would still be required. In this case, the current study suggests some triaging strategies to identify and prioritise the CYP who would most benefit from a face to face appointment. These include: patients with pre-existing concern about their physical well-being such as weight already below 9^th^ centile, those on the upper ceiling of the normal range of medications (e.g., 2.1mg/kg of methylphenidate), patients who had had home measurements that were outside normal limits, or those with other presentations considered to require urgent face to face clinical review.

The strengths of this study include the use of both quantitative and qualitative comments to explore the experiences of the Paediatricians who are supporting CYP with ADHD. Another strength is the UK wide membership of GSF, which provides some support for the external validity in relation to neurodevelopmental paediatricians' practice during the period of the survey. Some implications for practice include rapid adoption of telemedicine, pragmatic application of clinical guidelines, maintaining some face to face treatment based on clinically triaged needs, and cross-service collaborations. The study's limitations include the relative small sample size, the absence of comparative pre-pandemic data on ADHD-related telemedicine, lack of data on non-ADHD paediatric practices, lack of location-specific data, and the cross-sectional and self-report nature of the survey.

## 5. Conclusion

The Covid-19 pandemic has had adverse effect on many CYP with ADHD and caused huge disruption to the ADHD services that support them. The pandemic continues to be a challenge through the emergence of new variants such as Omicron that was causing the re-introduction of restrictions at the time of writing this paper. Thus, it is possible that disruptions to ADHD services are likely for some time. This shows the need to continue to learn from helpful service adaptions that can be maximised to provide more stable and sustainable ADHD services during this pandemic. Also, ADHD services need to consider continuing positive pandemic-engendered ADHD service changes that are now emerging from the literature where such changes can help to improve effective and efficient care delivery even after the current pandemic. These positive changes that are now emerging from the literature could also serve as blue prints in the event of a future pandemic.
